# Tailored Communication Within Mobile Apps for Diabetes Self-Management: A Systematic Review

**DOI:** 10.2196/jmir.7045

**Published:** 2017-06-23

**Authors:** Heidi Holmen, Astrid Klopstad Wahl, Milada Cvancarova Småstuen, Lis Ribu

**Affiliations:** ^1^ Department of Nursing and Health Promotion Faculty of Health Sciences Oslo and Akershus University College of Applied Sciences Oslo Norway; ^2^ Department of Health Sciences Institute of Health and Society, Faculty of Medicine University of Oslo Oslo Norway

**Keywords:** diabetes mellitus (MeSH), communication (MeSH), mobile apps, self-management, systematic review, mHealth

## Abstract

**Background:**

The prevalence of diabetes is increasing and with the requirements for self-management and risk of late complications, it remains a challenge for the individual and society. Patients can benefit from support from health care personnel in their self-management, and the traditional communication between patients and health care personnel is changing. Smartphones and apps offer a unique platform for communication, but apps with integrated health care personnel communication based on patient data are yet to be investigated to provide evidence of possible effects.

**Objective:**

Our goal was to systematically review studies that aimed to evaluate integrated communication within mobile apps for tailored feedback between patients with diabetes and health care personnel in terms of (1) study characteristics, (2) functions, (3) study outcomes, (4) effects, and (5) methodological quality.

**Methods:**

A systematic literature search was conducted following our International Prospective Register of Systematic Reviews (PROSPERO) protocol, searching for apps with integrated communication for persons with diabetes tested in a controlled trial in the period 2008 to 2016. We searched the databases PubMed, Medical Literature Analysis and Retrieval System Online (MEDLINE), Cumulative Index to Nursing and Allied Health Literature (CINAHL), Cochrane Central, Excerpta Medica database (EMBASE), ClinicalTrials.gov, and the World Health Organization (WHO) International Clinical Trials Registry Platform. The search was closed in September 2016. Reference lists of primary articles and review papers were assessed. The Preferred Reporting Items for Systematic Reviews and Meta-Analyses (PRISMA) guidelines were followed, and we applied the Cochrane risk of bias tool to assess methodological quality.

**Results:**

We identified 2822 citations and after duplicate removal, we assessed 1128 citations. A total of 6 papers were included in this systematic review, reporting on data from 431 persons participating in small trials of short duration. The integrated communication features were mostly individualized as written non–real-time feedback. The number of functions varied from 2 to 9, and blood glucose tracking was the most common. HbA_1c_ was the most common primary outcome, but the remaining reported outcomes were not standardized and comparable. Because of both the heterogeneity of the included trials and the poor methodological quality of the studies, a meta-analysis was not possible. A statistically significant improvement in the primary measure of outcome was found in 3 of the 6 included studies, of which 2 were HbA_1c_ and 1 was mean daytime ambulatory blood pressure. Participants in the included trials reported positive usability or feasibility postintervention in 5 out of 6 trials. The overall methodological quality of the trials was, however, scored as an uncertain risk of bias.

**Conclusions:**

This systematic review highlights the need for more trials of higher methodological quality. Few studies offer an integrated function for communication and feedback from health care personnel, and the research field represents an area of heterogeneity with few studies of highly rigorous methodological quality. This, in combination with a low number of participants and a short follow-up, is making it difficult to provide reliable evidence of effects for stakeholders.

## Introduction

About 415 million people have diabetes globally, and management of diabetes and its complications remains a global health emergency that already accounts for 12% of global health expenditure [1,2]. Diabetes’ impact is related to micro- and macrovascular complications [3,4] as well as deteriorated quality of life and increased rates of depression and anxiety [5,6]. The mobile health (mHealth) literature indicates that individuals using mobile apps for self-management achieve positive health outcomes [7]. Within the diabetes literature, both beneficial and adverse effects of mHealth solutions for self-management have been discussed; in summary, apps may be feasible and convenient for many but not all because of preferences, economy, and health literacy [8-11]. Possible functions in mobile apps include interaction functions such as messages and chatting with health care personnel (HCP); health-monitoring functions such as tracking blood glucose, weight, blood pressure, and medication; lifestyle-monitoring functions like physical activity and dietary habits; and educational functions supplying information. In addition, tracking of psychosocial status using patient-reported outcomes (PROs) is recognized as important in improving the understanding of living with a chronic disease and quality of care [12,13]; however, this function remains rare in apps for diabetes [14].

We argue that a key limitation of previous reviews is their lack of specific focus on communication, despite the emphasis Chomutare and colleagues [15] have placed on personalized education and feedback. The possibility for patients and HCP to review patient data within an app has been discussed previously [9,11,16,17]; however, it has not been thoroughly investigated. This is similar to a discussion we have had in our previous research after testing a mobile diabetes diary app with or without telephone contact with a diabetes specialist nurse, where the diabetes specialist nurse did not review any patient-related data within the app [18-20]. Despite encouragement, the participants did not discuss their data during their HCP consultations. Subsequently, some of our patients emphasized that if HCP had monitored, reviewed, or given feedback on their data through the app, the positive contributions of their data tracking and health counseling might have been greater (personal communication by Astrid Torbjørnsen, November 18, 2016). Further, our participants had a high disease burden and an undebatable need for change [21], so their needs were not met in our low-intensity intervention, and feedback based on the individual patient data might have changed this. In addition, Chomutare and colleagues [15] revealed a lack of personalized feedback in the apps they reviewed in 2011, and argued that this might be the missing link in diabetes self-management supported by apps. In general, earlier reviews of mobile apps for diabetes self-management include both reviews of apps available commercially evaluating mainly content and user ratings [10,14,15,22] and reviews of research and controlled trials to investigate possible effects of apps [7,23]. To date, there seems to be limited but encouraging evidence for the effectiveness of such apps compared with usual care, but the lack of rigorous research methodology is a weakness [9].

Within the research on technology-supported self-management, the effects of HCP communication via short message service (SMS), either alone or in combination with apps, have been investigated and have demonstrated promising results in the reviewed literature [24-26]. These effects might increase when the communication function is integrated within the app. Communication between patients and HCP based on individual health data to support the self-management of diabetes may produce improved health outcomes [27] such as increased self-management skills [18,20], increased self-monitoring of blood glucose and foot inspections, and decreased hemoglobin A_1c_(HbA_1c_) [28], as well as increased self-management and satisfaction with care with decreased diabetes distress and body mass index (BMI) [29]. Further, this tailored communication has been suggested to be a key preference among patients and providers [9,15]. Several professions might be involved: primary care physicians, diabetes specialist nurses, podiatrists, endocrinologists, clinical nutritionists, and others. Earlier research has suggested that alarmingly few patients attend self-management programs [30], and travel distances, rural areas, costs, and more might compromise the frequency of face-to-face HCP consultations, where technology might be an efficient alternative [8,23]. Receiving feedback on how to self-manage could represent a better solution for the patient than gathering data and reviewing them alone, and this would make the app more valuable than a paper-based diary [8]. Patients are increasingly becoming consumers of health, and if persons with diabetes prefer to communicate with their HCP through an app, it remains to be investigated whether apps with tailored communication can support diabetes self-management.

Self-management interventions have traditionally been based on theoretical frameworks, which are necessary to understand change [31]. Further, there has been proposed a linearity between applied theory and effect [32]. However, as mHealth becomes more frequent, a lack of theoretical foundation has been pointed out [8,9]. The goal of several apps is to help promote behavior change, which supports the argument for theory-based interventions. A recent review describes the need for integrating cognitive behavioral therapy into apps for diabetes, where the authors also propose a framework to reach this goal [33], which is an important step forward in understanding behavior change supported by mHealth and further increasing the quality of the apps.

Research on mobile apps with an integrated, tailored communication function is scarce, as the app interventions often include additional phone calls [34], SMS [35], face-to-face meetings [36], group meetings, or some combination of these [37,38] in addition to the mobile app itself. To the best of our knowledge, results based on apps with integrated and tailored communication alone have not been systematically summarized. This review aims to address this knowledge gap by systematically reviewing studies that aimed to evaluate integrated communication within mobile apps for tailored feedback between patients with diabetes and HCP in terms of (1) study characteristics, (2) functions, (3) study outcomes, (4) effects, and (5) methodological quality.

## Methods

### Protocol and Registration

The review protocol [39] was registered in the International Prospective Register of Systematic Reviews (PROSPERO) [CRD42016038640] and was presented and discussed by the first author in an oral conference presentation [40] in accordance with the PROSPERO [41] and the Preferred Reporting Items for Systematic Reviews and Meta-Analyses Protocols (PRISMA-P) [42,43].

### Information Sources and Search

A systematic literature search was conducted according to the PRISMA guidelines [44]. Medical literature published from January 2008 was searched in January 2016, with an updated search closed on September 23, 2016, using Medical Literature Analysis and Retrieval System Online (MEDLINE) , PubMed, Cumulative Index to Nursing and Allied Health Literature (CINAHL), Excerpta Medica database (EMBASE), ClinicalTrials.gov, and the World Health Organization (WHO) International Clinical Trials Registry Platform. We reviewed reference lists of relevant reviews and studies, and we also conducted hand searches in relevant journals of the field in addition to studies based on tips from colleagues in the field.

In collaboration with a librarian at the Oslo and Akershus University College of Applied Sciences and a librarian at the University in Oslo, we organized a search strategy consisting of the terms “mobile applications,” “cell phones,” “mobile phones,” “smartphones,” “portable applications,” “mobile technology,” “portable technology,” or “app.” These were then combined with “diabetes mellitus” and/or “diabetes mellitus type 1” and/or “diabetes mellitus type 2” and/or “diabetic ketoacidosis.” The search strategy was tailored to each database for optimal results (Textbox 1). The specific search strategy for each database can be provided by the first author upon request. We did not set a language limitation; however, we did set a limitation on publication year to studies published from 2008, as we decided technologies prior to 2008 were unlikely to be mobile apps.

### Eligibility Criteria

To be eligible, a study had to test a mobile app (software in a smartphone) with an integrated communication function for communication and/or feedback between patients and providers based on individual patient data. In this review, communication is conceptualized as medically trained personnel providing any kind of feedback based on patient data, being real time, chatting, individualized algorithms, or individualized trend analyses. The patient group had to have diabetes and be over the age of 16 years. The trials had to have a control group, either randomized, quasirandomized, or controlled clinical trial. We excluded trials that were for the primary prevention of diabetes, those regarding gestational diabetes, and those pertaining to a closed-loop or artificial pancreas system, as we regard those individuals to be unique in the way they perceive change and interact with HCP.

**Search strategy applied in MEDLINE.**

Search strategy:

1. Mobile applications/ (697)
2. Cell phones/ (5888)
3. (Smartphone* or smart phone* or mobile phone* or cell phone* or cellphone*).tw.kf (7888)
4. (Mobile adj3 application*).tw.kf (1077)
5. (Portable adj3 application*).tw.kf (276)
6. (Mobile adj3 technolog*).tw.kf (1322)
7. (Portable adj3 technolog*).tw.kf (161)
8. (App or apps).tw.kf (15895)
9. Or/1-8 (26696)
10. Diabetes mellitus/ or exp diabetes mellitus, type 1/ or exp diabetes mellitus, type 2/ or diabetic ketoacidosis/ (246647)
11. Diabetes.tw.kf (386565)
12. 10 or 11 (448207)
13. 9 and 12 (643)
14. Limit 13 to yr=“2008-current” (565)

### Study Selection

Two reviewers (HH and LR) independently reviewed all the titles and/or abstracts from the search. We applied our inclusion and exclusion criteria set a priori. For possibly eligible studies, a full text copy was retrieved and reviewed independently by HH and LR. Discrepancies were resolved by discussion or with the involvement of a third reviewer (AKW). Authors were contacted consecutively to clarify study design and determine whether the intervention was an app with integrated and tailored communication and no additional communication components. We sent one reminder to the nonresponders.

### Data Extraction

Data were extracted for all eligible studies using a structured form that included descriptive information, type of design, outcomes, and follow-up with results and dropouts, as well as any data regarding a theoretical framework or a guideline-based approach in the app development or feedback process of the intervention. One reviewer (HH) performed the extraction, while a second reviewer (LR) performed quality assurance and checked that correct information was collected.

The baseline characteristics of the included trials are reported as means from the original papers and as weighted means to summarize overall sample characteristics of this systematic review. When a weighted mean is given, a median is not reported as there were small discrepancies between weighted means and medians.

### Outcomes

In this systematic review, we reviewed diverse health outcomes (physical and psychosocial) used as both primary and secondary outcomes.

### Quality Assessment

The information reported in each article was used to assess the methodological quality of each study using the Cochrane methodology for risk of systematic bias (ROB) [45]. The ROB scoring was performed individually by 3 researchers (HH, AKW, and LR) and discussed to achieve consensus. To systematize the risk scores, Review Manager (Cochrane Community) was used.

**Figure 1:**
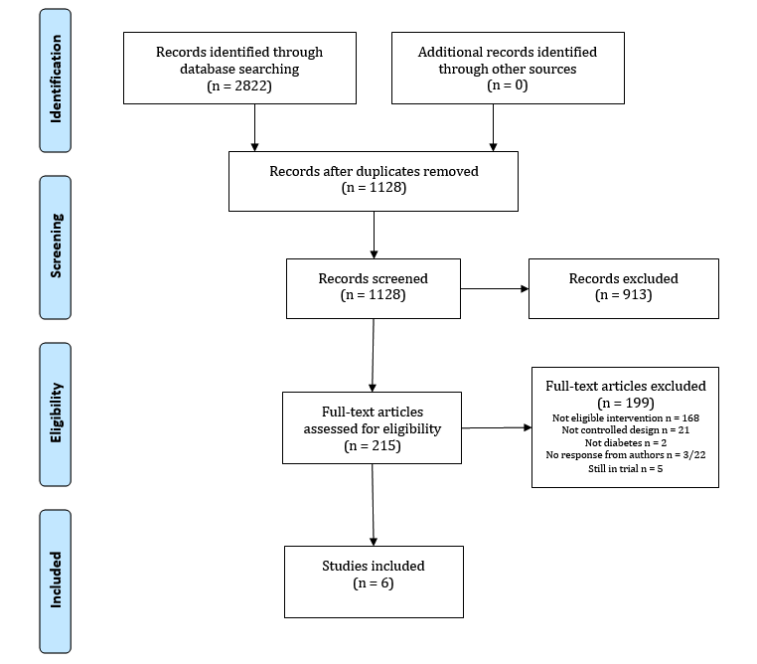
Flowchart.

## Results

### Summary

A total of 2822 papers were identified during the search (Figure 1). After the removal of 1694 duplicates, the remaining 1128 citations were screened through title and/or abstract, and we removed 913 citations because they clearly did not meet our inclusion criteria. The full text of the remaining 215 citations was then obtained to clarify their study details, and we contacted 22 authors to clarify that their intervention consisted of an app with integrated and tailored communication and no additional contacts. Of the 22 authors we contacted, 18 responded immediately, 1 responded after a reminder, and 3 requests remained unanswered after 1 reminder. The corresponding citations were excluded from the review. After the termination of the search, 6 citations [46-51] were included in this review. The main reasons for exclusion were research related to the prevention of diabetes, mobile apps without communication, and other media used for communication including email, phone calls, and SMS (texting). Several studies were identified that had an intervention consisting of a mobile app with communication, while some of these had additional contacts by telephone, Web, or face to face and were therefore excluded [34-38].

### Study Characteristics

The included studies were heterogeneous in study procedures and design (Tables 1 and 2); 4 used a randomized controlled trial (RCT) design, of which 1 was a pilot study. One study used a matched, controlled design, and 1 study randomly selected participants before assigning them into 2 groups. A total of 3 of the studies were conducted in Asia, (ie, Japan [50], China [51], and Korea [47]), 1 in the Democratic Republic of Congo [49], 1 in Canada [48], and 1 was a multicenter study conducted in 3 European countries [46]: Italy, Spain, and the Czech Republic. The papers were published between the years 2012 and 2016. Usual care was not described in detail in any of the included studies.

**Table 1:** Study characteristics.

Study	Year	Country	Randomization	Allocation	Dropouts n (%)
Fioravanti [46]	2015	Czech Republic, Italy, Spain	Not described	Unclear	3 (5.6)
Kim [47]	2014	Korea	Matched control design, not randomized	Unconcealed	3 (7.9)^a^
Logan [48]	2012	Canada	Block randomization using blocks of 4 and 6	Unclear	6 (5.5)
Takenga [49]	2014	Congo, Germany	Not described	Unclear	9 (22.5)
Waki [50]	2014	Japan	Not described	Unclear	5 (9.3)
Zhou [51]	2016	China	Random number table	Unclear	NA^b^

^a^Dropout only from the intervention group.

^b^NA: not available.

**Table 2:** Trial design.

Study	Patients included	Intervention group	Control group	Duration	Measurement times
Fioravanti [46]	51^a^	METABO app, chatting and message function with HCP^b^	Usual care	1 month	Baseline and 1 month
Kim [47]	70^c^	Mobile smartcare app, weekly feedback from HCP, warnings when hypos registered, and reminders	Matched controls from electronic medical records	3 months	Baseline and 3 months
Logan [48]	110	Real-time self-management messages based on care paths of averages of transmitted blood pressure readings	Tele monitoring without messages	12 months	Baseline and 12 months
Takenga [49]	40	MobilDiab app, feedback and messages from HCP	Usual care	2 months	Baseline and 2 months
Waki [50]	54	Dialbetics app, feedback based on patient data and guidelines	Usual care	3 months	Baseline and 3 months
Zhou [51]	100	Welltang app, weekly feedback, and upon patient-request	Usual care, monthly	3 months	Baseline and 3 months

^a^Included N=54, numbers given for N=51 completers.

^b^HCP: health care personnel.

^c^Included N=73, numbers given for N=70 completers.

**Table 3:** Participant characteristics at baseline of included trials, N=6.

	Age (mean years)	Gender (male/female)	Type 1 diabetes	Type 2 diabetes	Duration of diabetes (mean years)	HbA_1c_^a^ (mean %)	BMI^b^ (mean kg^2^)
Fioravanti [46] N=51^c^	48.0^d^	36/15^d^	29^d^	22^d^	20.0^d^	7.9^d^	25.7^d^
Kim [47] N=70^e^	52.8	40/30	0	70	11.8	7.7	25.0
Logan [48] N=110	62.9	61/49	NA^f^	NA	NA	7.4	30.9
Takenga [49] N=40	53.3	29/11	NA	NA	NA	8.6	NA
Waki [50] N=54	57.2	41/13	0	54	9.0	7.0	26.7
Zhou [51] N=100	54.2	57/43	18	82	6.6	9.8	23.0

^a^HbA_1c_: hemoglobin A_1c_.

^b^BMI: body mass index.

^c^Included N=54, numbers given for N=51 completers.

^d^Provided upon request.

^e^Included N=73, numbers given for N=70 completers.

^f^NA: not available.

### Participants

Overall, the 6 trials reported data from 431 participants as shown in Table 3, with a sample size varying from N=40 to N=110 and a median of 64 participants. One trial did not give any demographic data in the original article [46]; however, the author provided this information upon request. A total of 2 trials did not report any data regarding their total of n=6 dropouts [46,47]. Overall, 47 participants were specified to have type 1 diabetes and 228 type 2 diabetes, while 2 studies [48,49] did not specify type of diabetes for their combined total of 150 participants. Weighted mean age was 55.8 years, including 264 males and 160 females. Duration of diabetes was provided upon request from one trial [46], and reported in 3 papers, giving a weighted mean of 11 years [47,50,51]. HbA_1c_ was collected in all trials [46-51] with a weighted mean of 8.1%, and BMI was reported in 4 papers [46,47,50,51] and provided by 1 author in an email, giving a weighted mean BMI of 26.5 kg^2^.

### Functions of the Mobile Apps

The mobile apps used in the included studies varied in their form and functions (Table 4), and a theoretical foundation was largely lacking.

The feedback used was either automatic or manual feedback, both tailored, and 4 apps also offered direct messages from the patient in free text. A total of 3 studies had automated individualized feedback consisting of text tailored to the participant baseline data and their current readings [46,48,50]. One of these had the participant data evaluated according to diabetes treatment guidelines [50], a second study had an additional message function [46], while a third had no additional feedback or messages [48]. The other 3 studies had individualized feedback given directly by the physician [49], medical staff [47], or the study team [51], and 2 of these had an additional message function for questions in free text [49,51].

The MobilDiab study had therapy plans, instructions, and recommendations sent by the physician in the app [49]. The Welltang app offered answers to questions within the day in addition to weekly or fortnightly feedback [51]. The METABO app [46] had both the app and the content of messages tailored to the type of diabetes: those with type 2 diabetes had a less complex app and received more persuasive messages; patients could also turn off alerts they did not want to receive and tailor the timing of the messages. DialBetics was an extensive app, consisting of automatic transfer of data and feedback based on blood glucose readings, diet, blood pressure, physical activity, and weight, where the users received immediate feedback based on every registration in the app, evaluated following the Japanese Diabetes Society guidelines [50].

A total of 2 apps had critical alerts sent to the patients if their entered readings were outside preset thresholds [46,51]. In MobilDiab, the physicians received an alert if emergency values were recorded and they instructed the patient [49], while in DialBetics, any readings outside preset thresholds triggered an alert sent to the study team [50]. A total of 3 apps alerted patients when they missed readings [48,50,51] and a fourth had automatic alerts regarding hyperglycemia; the medical team called the patient if they recorded a hypoglycemic value or if they missed several readings [47].

**Table 4:** Functions of the mobile apps.

Study	Communication	Blood glucose	Diet	Blood pressure	Medication	Physical activity	Weight
Fioravanti [46]	Chat with HCP^a^, messages and individualized automated feedback according to the TTM^b^	Manual input	Manual input	Manual input	Manual input	Manual input	Manual input
Kim [47]	Messages and individualized feedback	Manual input	—	Manual input	—	—	—
Logan [48]	Individualized automated feedback	—	—	Bluetooth	—	—	—
Takenga [49]	Messages and individualized feedback	Automatic transfer and manual input	Manual input	Manual input	Manual input	Manual input	Manual input
Waki [50]	Individualized automated feedback according to Japan Diabetes Society guidelines	Automatic transfer	Voice, text, or photo of meal	Automatic transfer	—	Automatic transfer of pedometer or voice or text	Automatic transfer
Zhou [51]	Messages and individualized feedback	Manual input	Manual input	—	Manual input	—	—

^a^HCP: health care personnel.

^b^TTM: transtheoretical model stages of change.

The most frequent function besides communication was registration of blood glucose; this was found in 5 apps. One of the 5 offered automatic transfer [50] of blood glucose readings from the meter to the app, while 3 had manual input of the measured blood glucose [46,47,51]. One app had both: automatic transfer from a specific glucose meter and manual input if the patients used a different meter [49]. Blood pressure measurement was offered in 5 of 6 apps, while diet and graphical trends of measures were offered in 4 out of 6 apps. Tracking and imputation of medication, levels of physical activity, and weight were functions in 3 out of 6 apps, in addition to their diabetes information functions. A total of 2 apps offered individual goal setting: 1 offered a connection to continuous glucose monitors and 1 had laboratory data in the app. None of the apps in the included studies had psychosocial measures as a function. The number of functions in addition to communication ranged from 2 to 9 with a median of 6 functions. In 3 of the included studies, the intervention also consisted of a digital solution like an app or a Web page for the involved HCP [46,49,51].

### Outcomes and Effects

Primary outcomes were specified in 5 trials, whereas 1 study [49] did not specify the order of the outcomes (Table 5), and various outcomes were used to evaluate the interventions in the individual trials.

HbA_1c_ was reported in 4 of the 6 included trials, and stated as the primary outcome in 3 papers. A total of 2 papers [50,51] reported a significant decrease in the intervention groups compared with the control groups, namely –0.4% and –1.95%, while 2 trials reported no change between groups [47,49] and the remaining 2 papers did not report change in HbA_1c_ as an outcome [46,48].

Change in blood pressure as an outcome was reported inconsistently using both systolic blood pressure (SBP), diastolic blood pressure (DBP), and mean blood pressure among the papers reporting blood pressure [47,48,50,51]. One paper reported changes in mean daytime ambulatory SBP as the primary outcome and found a significant decrease in the intervention group compared with the control group [48]. A total of 3 papers reported no significant change in either SBP or DBP between the intervention and control groups [47,50,51], while 2 papers reported no measures of blood pressure [46,49].

Regarding diabetes knowledge, there were no significant differences between the intervention group and the control group in 2 trials using this as an outcome [46,51], although neither used validated measures in their data collection.

Various assessments of usability and satisfaction were reported [46,47,49-51] but common for all was the use of nonvalidated and comparable questionnaires for this evaluation.

One paper reported a significant increase in depressive symptoms using the Hospital Anxiety and Depression Scale (HADS) in the intervention group [48].

**Table 5:** Outcomes and effects of included studies.

Study	Outcome measures^a^	Effects			
		HbA_1c_^b^	Blood pressure	App-related evaluations	Other evaluations
Fioravanti [46]	Feasibility (primary), acceptance, adherence, usage, knowledge, glycemic control, quality of life	NA^c^	NA	Feasible	Increased medication adherence and diabetes knowledge in intervention group
Kim [47]	HbA_1c_ (primary), anthropometrics, satisfaction, comfort, convenience, functionality	No change	SBP^d^increased in intervention group; not significant between groups	Increased satisfaction	NA
Logan [48]	Mean daytime ambulatory SBP (primary), antihypertensive medication, HADS^e^, comfort with home BP^f^measurement	NA	Significant decrease in mean daytime ambulatory SBP	NA	Worsened HADS in intervention group
Takenga [49]	HbA_1c_, mean blood glucose, usability, acceptance, efficiency, therapy satisfaction	Decreased in intervention group	NA	Positive usability	NA
Waki [50]	HbA_1c_ (primary), fasting blood glucose, BP, BMI^g^, LDL^h^, HDL^i^, triglycerides, medication, self-management, usability	Significant decrease in intervention group	NA	Positive usability	NA
Zhou [51]	HbA_1c_ (primary), blood glucose, LDL, weight, BP, hypoglycemia, satisfaction with diabetes care, usability of app, diabetes knowledge, self-care	Significant decrease in intervention group	No change	Positive usability (dichotomous)	Significant increase in diabetes knowledge and self-care in the intervention group

^a^Questionnaire not standardized unless stated otherwise.

^b^HbA_1c_: hemoglobin A_1c_.

^c^NA: not available.

^d^SBP: systolic blood pressure.

^e^HADS: Hospital Anxiety and Depression Scale.

^f^BP: blood pressure.

^g^BMI: body mass index.

^h^LDL: low density lipoprotein.

^i^HDL: high density lipoprotein.

### Methodological Quality Assessment

**Figure 2:**
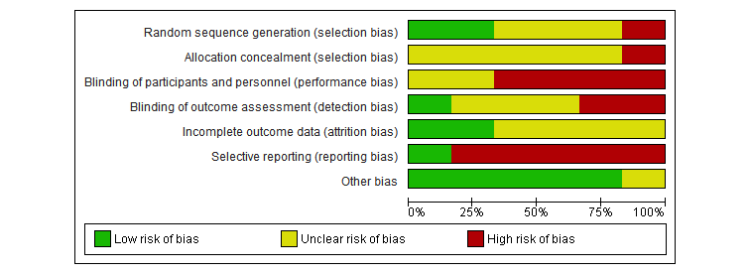
Risk of bias: review authors’ judgements about each risk of bias item presented as percentages across all included studies.

**Figure 3:**
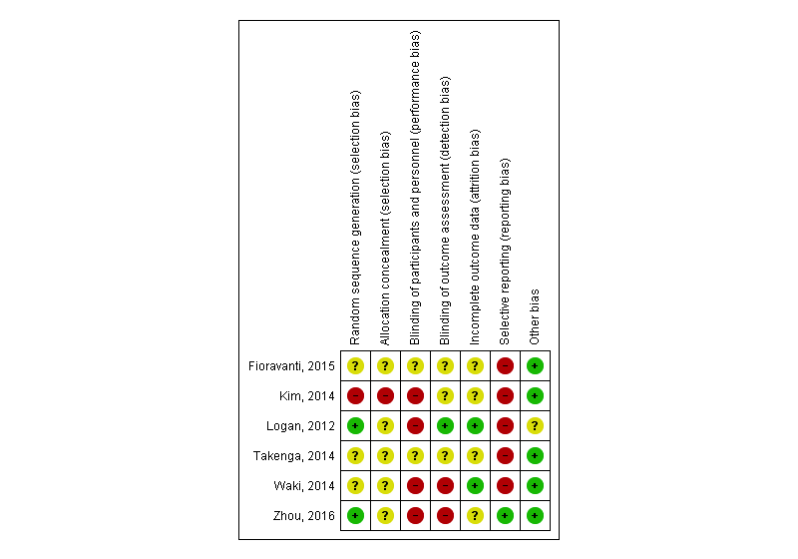
Risk of bias summary: review authors’ judgments about each risk of bias item for each included study.

Overall, the methodological quality as assessed by the ROB [45] was dominated by uncertainty risk because of lack of information in the included articles, as visualized in Figures 2 and 3. A lack of information in the publications was scored as “uncertain,” while we rated articles with sufficient information according to the Cochrane ROB guidelines [45]. As Figure 2 shows, the overall risk of bias is greatest regarding performance bias and selective reporting because of lack of blinding of the intervention and lack of reporting outcomes a priori in databases such as ClinicalTrials.gov or publishing the research protocols. “Other bias” is the domain with lowest risk, but several points can be highlighted, including economic interests, patent interests, and other factors influencing free research. We have, however, no indication that such issues are present in our included studies and have rated them low.

When we applied the ROB tool to our 6 papers, “unclear” was given 18 times, “high” was given 11 times, and “low” was given 11 times, supporting an overall unclear ROB among the included studies. Randomization procedures were reported insufficiently in 3 papers [46,49,50], resulting in an unclear ROB, while Kim and colleagues [47] had a matched control design and hence had a high risk because the participants were fully aware of their group. Blinding of participants and personnel was not performed in 4 papers [47,48,50,51], leading to a high risk according to guidelines, and not mentioned in 2 papers [46,49], giving an unclear risk. Blinding of the outcome was performed in 1 study giving a low risk of bias [48]. The 2 trials reporting not having performed blinding of outcomes [50,51] were scored as high risk, and the 3 papers [46,47,49] not mentioning this were scored as unclear risks. The completeness of outcome data was unclear in 4 of the 6 papers as there were inconsistencies in reporting rates and reasons for attrition; 2 papers, however, reported sufficient information and were given a low risk of attrition bias. Selective reporting was assessed as high in all 5 trials not registered in a clinical trials database and uncertain when this information was lacking. One study was registered in a WHO-approved register for clinical trials and hence scored low on reporting bias [51].

## Discussion

### Principal Findings

This systematic review describes the study characteristics, functions, outcomes, effects, and methodological quality of intervention trials studying apps for diabetes self-management with a tailored and integrated HCP-patient communication function. To summarize, the studies included in this review represented a heterogeneous research area. The mobile app communication functions integrated in the studied apps were largely automated feedback from HCP, and the number of additional functions varied from 2 to 9, of which blood glucose registration was the most frequent. Statistically significant effects were found in 3 of the 6 trials: 2 reported a decrease in HbA_1c_ [50,51] and 1 reported a decrease in SBP [48]. The unclear methodological quality of these few studies has implications for the evidence from this systematic review. We argue, however, that our paper has an important message regarding the state of the research field, and it highlights the need for more controlled trials of higher methodological quality.

We found only 6 controlled trials with apps offering integrated communication functions, and SMS, phone calls, and face-to-face consultations are still common in the mHealth research field. From this rigorous yet wide systematic search, we had anticipated a larger number of controlled trials investigating mobile apps with individualized and integrated feedback from HCP, as a 2011 review called for such research [15] and the availability of hundreds of apps is frequently highlighted [9,23]. Previous research has also discussed the possibility of communication through a mobile app for health purposes (eg, for collecting and analyzing health data) related to the idea of one platform to serve all patient needs [7,11]. We identified several apps through our search, but their interventions were not in the scope of this review, with additional contacts through either email, SMS, or phone calls [34-38]. Further, we might have had a higher number of eligible trials if the search had included other chronic conditions. However, as diabetes self-management might be uniquely complex, including clinical variables, we suggest that the results derived from such research would have been of less value to the diabetes field.

Others have also discussed whether apps are less scientifically tested than other medical solutions [9], possibly explaining the low number of relevant scientific papers for this review. It might be difficult to commercialize an app involving HCP because of the practicalities and high costs. Further, it is increasingly recognized that apps should be regulated in terms of their effect, security, and privacy [9,23,52]. Currently, the US Food and Drug Administration offers unbinding guidelines for their regulation, approval, and clearance of apps, without the control authority [53]. CE marking is frequently applied in the European countries, however, this is based on self-certification and accounts for the health, safety, and environment protection related to the app. A third regulation is the Health Insurance Portability and Accountability Act (HIPAA), which pertains to the US national standards for electronic health, especially regarding devices that collect, store, or share identifiable data with HCP. None of the current reviewed papers discussed these regulations. Navigating these regulations, in addition to cost and practicalities, might make research in this area less attractive because the real world demands cost effectiveness [23,47], an outcome not covered by this review.

The studies included in this review include research from Northern America, Europe, Asia, and Africa, and all have small samples and short follow-ups. The longest follow-up was 12 months [48], whereas the rest had 1 to 3 months, possibly too short to prove an effect as one author suggested [47]. As is common in the area, the majority of the studies did not distinctly separate type 1 and type 2 diabetes, except for one [46], describing a less complex intervention for those with type 2 diabetes. We argue that although persons with diabetes experience many of the same symptoms and must take the same measures, it would have been of interest to investigate the 2 diabetes groups separately, as the psychological aspect and the person’s interest in change and self-management may differ.

The most common function besides communication appeared to be self-monitoring of blood glucose, a function in 5 of the 6 apps. This is not surprising, as self-monitoring of blood glucose is found to lower HbA_1c_ [54]. However, it is still debated whether persons with type 2 diabetes benefit from blood glucose measures [55]. Additional functions are crucial, as an app should offer more than the traditional paper diary [8]. It is alarming that few developers of apps discuss behavior change theory or treatment guidelines, even though the majority of available apps, including those in this review, aim to change behavior or habits [15,22]. Treatment guidelines or behavior change theory should guide intervention development as both can increase the quality of the app [7,8,9], and possibly this is best achieved if researchers from several fields work together (eg, health care researchers and technological engineers). Further, a linearity between behavior change theory and effects has been suggested [32], and its use would strengthen all arguments regarding the practical use of the app. One example may be the input of values, where greater personal reflection is gained through manual input [35]. However, manual input can be time consuming and there is a larger risk of faulty input than with automatic input. As pointed out in 2 of the current trials [48,50], tracking of blood pressure might reveal those with an out-of-range blood pressure in need of medication that might not be identified in a general practitioner office visit. The same argument is valid for monitoring blood glucose values: thus, these 2 functions of blood glucose and blood pressure remain important to reach the treatment goals for diabetes.

Another point of interest is that medication tracking was a function in just 3 of the 6 apps [46,49,51], meaning that the individualized feedback in the remaining 3 apps [47,48,50] does not evaluate usage of medication that might be critical for the patients. Nor was there tracking of psychological measures in the 6 trials we included. Measures of this kind are not much used in apps for diabetes; however, they might provide useful information for the patient and the provider [33]. For example, depressive symptoms are a significant risk in those with diabetes [6]. There is a known association between anxiety and self-focus on bodily symptoms [48] that may be triggered by self-monitoring of blood glucose, and this may support the need for measures to reveal such symptoms as they can degrade self-management and glycemic control.

Several outcomes were used to evaluate the apps’ ease of use among the studies in this review: patient and/or HCP satisfaction, degree of technical issues like delay of data transfer, use of time, acceptance of feedback, and usefulness. However, as none of these used validated measures, the evidence regarding app-related evaluations such as satisfaction, acceptability, usability, and feasibility as outcomes from this systematic review is weak. These concepts will, however, remain important to ensure that the apps are used and 1 paper highlighted the association between use or satisfaction and effect [47]. As Kim and colleagues [47] argue in their paper, a well-functioning tool must be provided to increase use and satisfaction and to decrease the risk of deteriorating glycemic control.

As a more standardized outcome, HbA_1c_ was reported in 4 of the 6 trials in our systematic review, and this seems to be the most common outcome in diabetes trials regardless of intervention. Whether HbA_1c_ is an appropriate outcome in trials aiming for lifestyle change is a relevant question that we have debated previously [18], as has Garabedian [23].

The current included trials did not report lifestyle measures such as physical activity or dietary habits, making an evaluation of their effect on lifestyle difficult. None of the studies reported adverse events or safety as an outcome, except that Zhou et al [51] reported that in their trial they were infrequent in both groups. We regard the lack of focus on adverse events as an important weakness as there is a risk of hypoglycemia attached to the use of apps because of possible changes in medication or behavior.

A total of 3 studies found significant effects in their primary outcomes: 1 in SBP [48] and 2 in HbA_1c_ [50,51]. The remaining 3 studies remained inconclusive. One possible explanation might relate to the patients’ interest in data tracking and the recurrent reminders of having a chronic illness. This leads back to the identification of the individual’s interest in mHealth and also that it might be useful for some, but not all. Attitudes and intentions should be clarified for an app to be useful, regardless of functions [56]. Both researchers and clinicians must remember that patients often have limited interest in tracking their health, and for the app to be useful, there should be some clarification of the patients’ expectancies of the app, its usefulness, and possible adverse events [57].

The overall methodological quality of the included trials was low, with small samples and weak designs, which threatens the generalizability and reliability of the results. The lack of detailed description of the comparison group is a limitation among all of the included studies, and although the national guidelines are often used to define usual care, we cannot evaluate the content of the comparison group. Further, poor reporting of study details made efficient evaluation difficult and important details regarding group allocation, blinding, and preregistration in trial databases were unclear. Use of a standardized guideline for reporting, such as eHealth Consolidated Standards of Reporting Trials (eHealth CONSORT) [58], would have improved the reporting significantly, supported by earlier research confirming that adherence to such guidelines is low in medical informatics [59]. A pooled analysis was not possible because of the high ROB and heterogeneous outcomes. The ROB domains regarding blinding have been discussed among the authors, as blinding of such interventions is often difficult [60]; however, blinding of the outcome studied should be possible. The authors have discussed the domain “other” in ROB, and possibly reporting according to the eHealth CONSORT [58] could improve the reporting and evaluation of intervention trials. However, this is only applicable for randomized trials. A strength among the studies was the low dropout rates, which must be considered unusual compared with earlier research in this field [61].

### Limitations

This review has some limitations. We performed a systematic search using rigorous methods; however, as MeSH terms are still new in the field of technology in health research, the use of keywords might have contaminated our search. A 2014 consensus paper [62] stated that the term “app” should be used before “application,” which might positively influence the field in the years to come but to date this is still not frequently applied. Our search had a high N, closely related to such contamination. This review only assessed published trials, and we cannot rule out any publication bias. The inclusion of trials in this review was demanding as the interventions often had additional communication outside the scope of this review, but we cannot provide accurate numbers on how many studies this applies to, largely because of the heterogeneity among the excluded trials and lack of resources to handle this information systematically. However, we argue that the heterogeneity of the interventions represents the field and that there is still no consensus regarding preferred communication with HCP. Further, we did not assess the quality of the apps included in the current trials, as this was not in the scope of our work, and this might represent a limitation.

The application of the Cochrane ROB tool [45] may represent a limitation, as this tool might not be applicable in pragmatic technology trials, related to the previous discussion on blinding. Another possible threat in interventions and trials evaluating use of apps might be the less frequent usage of the app over time. Because of the short follow-ups, we cannot confirm whether this is a decline in use or whether it represents a more dynamic use of an app in periods where the persons with diabetes want to or should use the app more. However, if the app use changes substantially during the study period, assumptions might be drawn on the wrong basis, and none of the current trials included app use as an outcome, either in terms of which functions were mostly used or app use frequency through the study period (eg, in terms of number of times that apps were accessed). The health literacy aspect can also contribute, as participants’ use may decline if the intervention/app is too difficult to understand [10].

### Implications

This systematic review has not produced specific evidence for stakeholders regarding future decisions. We believe that the next generation of patients with diabetes has different needs and requests for the health care system and technology development and use than what is available today. Another important point might be the conflicting interests among the health care researchers and technology researchers regarding patent or economic interests in the device or app they are testing, and possibly, their drive for positive results could bias the published material. Therefore, it is even more important in this field to perform a thorough and unbiased evaluation and report the results from controlled trials regardless of their outcomes, which would form new evidence and provide benefit and information for policymakers. We suggest using a health technology assessment framework like the Model for the Assessment of Telemedicine (MAST) [63] for a scientific evaluation of important domains when testing technology and also the eHealth CONSORT for reporting scientific trials [58]. Further, the availability of hundreds of apps makes it difficult to find clinically relevant apps, and the need for updated reviews will continue to be large in the future [23]. There is, however, a need for higher methodological quality trials to improve the field and inform future reviews. The studies in this review were mostly pilot studies with small sample sizes and interventions that might be too extensive to be implemented in real-life contexts.

### Conclusion

The conclusions from this systematic review are limited. The unclear and poor methodological quality of this emerging research field is of major concern, and although 3 studies found that apps with integrated feedback significantly improve the primary outcome, the evidence has limitations because of its poor methodological quality. Mobile apps will be a part of the health care system in the future; therefore, we require robust research in this area to make the right choices for the patient, for the health care system, and for society.

## Abbreviations

BMI: body mass index
BP: blood pressure
CINAHL: Cumulative Index to Nursing and Allied Health Literature
CONSORT: Consolidated Standards of Reporting Trials
DBP: diastolic blood pressure
EMBASE: Excerpta Medica database
HADS: Hospital Anxiety and Depression Scale
HCP: health care personnel
HbA _1c_: hemoglobin A1c
HDL: high-density lipoprotein
LDL: low-density lipoprotein
MAST: Model for the Assessment of Telemedicine
MEDLINE: Medical Literature Analysis and Retrieval System Online
NA: not applicable
PRO: patient-reported outcome
PROSPERO: International Prospective Register of Systematic Reviews
PRISMA-P: Preferred Reporting Items for Systematic Reviews and Meta-Analyses Protocols
PRISMA: Preferred Reporting Items for Systematic Reviews and Meta-Analyses
RCT: randomized controlled trial
ROB: risk of bias
SBP: systolic blood pressure
SMS: short message service
TTM: transtheoretical model stages of change
WHO: World Health Organization
